# O.2.2-3 Virtual nature as an intervention to promote connectedness with and visitation of nature among university students: a randomized trial

**DOI:** 10.1093/eurpub/ckad133.118

**Published:** 2023-09-11

**Authors:** Elena Brambilla, Karen Stendal, Vibeke Sundling, Giovanna Calogiuri

**Affiliations:** Centre for Health and Technology, Department of Nursing and Health Sciences, University of South-Eastern Norway, Drammen, Norway; Department of Business, Marketing and Law, University of South-Eastern Norway, Ringerike, Norway; Department of Optometry, Radiography and Lighting Design, University of South-Eastern Norway, Konsberg, Norway; Centre for Health and Technology, Department of Nursing and Health Sciences, University of South-Eastern Norway, Drammen, Norway; Section for Public Health, Department of Public Health and Sport Sciences, Faculty of Social and Health Sciences, Inland Norway University of Applied Science, Elverum, Norway; Elena.Brambilla@usn.no

## Abstract

**Purpose:**

Human-nature interactions, including visitation of nature and nature connectedness (NC), a psychological construct defining an individual’s cognitive and affective connection with the natural world, are associated with a variety of health and well-being indicators. University students often experience high levels of stress and mental health challenges, while having little access to and few opportunities to interact with nature. This randomized controlled trial explored the effectiveness of a virtual nature intervention, delivered either through two-dimensional (2D) or immersive virtual reality (VR) devices, in eliciting increased NC and the likelihood of visiting a naturalistic location among university students.

**Methods:**

Thirty-eight university students (age 24.7±5.4 year) were randomized into two groups and viewed a video of a naturalistic location in the vicinity of the university campus either through VR or 2D devices. Further, participants received information about the naturalistic location and were invited to an organized hiking tour in that location. Pre- and post-assessment of NC, intention to engage in active nature visits, intention to visit the location viewed in VR or 2D, and intention to participate in the hiking tour were collected. Additionally, participation in the hiking tour was recorded.

**Results:**

A mixed between-within subjects ANOVA showed that both conditions significantly improved NC (F_1,35_ = 293.302; p < 0.001), intention to visit the location (F_1,36_ = 18.848; p < 0.001), and intention to participate in the hiking tour (F_1,36_ = 12.450; p < 0.001), but did not improve intention to engage in active nature visits, with no significant differences between the type of virtual nature exposures (VR vs. 2D). Six (16.7%) students participated in the organized tour, three from each condition. The type of virtual nature exposure was not a significant predictor for participating in the tour.

**Conclusions:**

This study provides evidence of the potential of virtual nature exposure in promoting NC and visitation of nature among university students, regardless of the technology employed (VR or 2D). More research is needed to better understand the impact of such types of intervention in supporting behavior changes and health in this population.

