# Correction to: O.2.2-3 Virtual nature as an intervention to promote connectedness with and visitation of nature among university students: a randomized trial

**DOI:** 10.1093/eurpub/ckad173

**Published:** 2023-09-29

This is a correction to: Elena Brambilla, Karen Stendal, Vibeke Sundling, Giovanna Calogiuri, O.2.2-3 Virtual nature as an intervention to promote connectedness with and visitation of nature among university students: a randomized trial, *European Journal of Public Health*, Volume 33, Issue Supplement_1, September 2023, ckad133.118, https://doi.org/10.1093/eurpub/ckad133.118

In the originally published version of this abstract, there was a typographical error in the surname of author Karen Stendal.

This has been corrected.

